# Emergence and Global Spread of Mpox Clade Ib: Challenges and the Role of Wastewater and Environmental Surveillance

**DOI:** 10.1093/infdis/jiaf006

**Published:** 2025-02-10

**Authors:** Ananda Tiwari, Thierry Kalonji, Taru Miller, Tim Van Den Bossche, Adriana Krolicka, Hypolite Muhindo-Mavoko, Patrick Mitashi, Marc Christian Tahita, Rolf Lood, Tarja Pitkänen, Vivi Maketa

**Affiliations:** Department of Food Hygiene and Environmental Health, Faculty of Veterinary Medicine, University of Helsinki, Helsinki, Finland; Microbiology Unit, Finnish Institute for Health and Welfare, Kuopio, Finland; National Monkeypox and Viral Haemorrhagic Fevers Control Program, Ministry of Health, Kinshasa, Democratic Republic of the Congo; Microbiology Unit, Finnish Institute for Health and Welfare, Kuopio, Finland; Infectious Disease Control and Vaccinations Unit, Finnish Institute for Health and Welfare, Helsinki, Finland; VIB-UGent Center for Medical Biotechnology, Ghent University, Ghent, Belgium; Department of Biomolecular Medicine, Faculty of Medicine and Health Sciences, Ghent University, Ghent, Belgium; Norwegian Research Centre, AS, Nygårdstangen, Bergen, Norway; Department of Tropical Medicine, Faculty of Medicine, University of Kinshasa, Kinshasa, Democratic Republic of Congo; Department of Tropical Medicine, Faculty of Medicine, University of Kinshasa, Kinshasa, Democratic Republic of Congo; Unité de Recherche Clinique de Nanoro, Institut de Recherche en Sciences de la Santé, Nanoro, Burkina Faso; Department of Clinical Sciences, Division of Infection Medicine, Faculty of Medicine, Lund University, Lund, Sweden; Department of Food Hygiene and Environmental Health, Faculty of Veterinary Medicine, University of Helsinki, Helsinki, Finland; Microbiology Unit, Finnish Institute for Health and Welfare, Kuopio, Finland; Department of Tropical Medicine, Faculty of Medicine, University of Kinshasa, Kinshasa, Democratic Republic of Congo

**Keywords:** Democratic Republic of Congo, epidemics, mpox clade Ib, wastewater-based surveillance

## Abstract

Several African countries, mainly the Democratic Republic of Congo, Burundi, and Uganda, are facing highly transmissible mpox clade Ib epidemics, prompting the World Health Organization to declare a Public Health Emergency of International Concern. It has spread to key travel hubs like Kinshasa, Bujumbura, and Kampala, increasing international spread risks. Current mitigation efforts focus mainly on medical care, diagnostics, vaccination, and infection prevention, but overlook wastewater and environmental surveillance (WES). WES can be effective in detecting hotspots and enabling rapid response through enhanced data collection and genomic sequencing. This perspective article reviews the latest outbreak situation and advocates integrating WES into response strategies.

## Teaser

This paper advocates for the integration of wastewater and environmental surveillance (WES) into mpox outbreak mitigation, emphasizing its potential in enhancing early detection and rapid response. WES complements existing measures in diagnostics, medical care, and infection prevention.

Mpox, formerly monkeypox, is a public health concern caused by the mpox virus (*Orthopoxvirus* genus, Poxviridae family). It spreads through contact with infected animals, humans, or contaminated materials [1]. The virus has 2 main clades: clade I (Congo Basin) and clade II (West Africa) [2]. Global attention surged after the 2022 multicountry clade IIb outbreak, currently reported in over 123 countries across Europe, North America, and Asia, with over 109 699 confirmed cases, and many deaths [3].

Mpox clade I cases (clade Ia and Ib) have surged in Africa, with 57 942 cases (46 794 suspected, 11 148 confirmed) and 1081 suspected deaths (1.8% case fatality rate) across African Union member states from 1 January to 3 November 2024 [3]. In the Democratic Republic of Congo (DRC), the outbreaks' epicenter, 39 501 suspected clade I cases (84.4%), including 8662 confirmed cases and 1073 suspected deaths [3]. Burundi and Uganda have emerged as recent hotspots, with 1726 and 359 confirmed cases, respectively [3]. From mid-September to 3 November 2024, cases increased by 19.0% in DRC, 59.7% in Burundi, and 90.3% in Uganda [3]. Other affected countries include Benin, Cameroon, Central Africa Republic, Congo, Egypt, Ghana, Liberia, Morocco, Mozambique, Nigeria, Rwanda, Sudan, and South Africa [3].

The DRC declared a national mpox epidemic on 18 December 2022 [4]. Currently, cases have surged across multiple provinces, including previously unaffected areas like Kinshasa, South Kivu, North Kivu, Lualaba, Kwango, Tanganyika, and Kongo-Central [5]. Mpox has now been reported in all 26 provinces, with the highest number of cases in Equateur, South Kivu, South Ubangi, and Sankuru (Table 1).

**Table 1.** Number of Suspected Cases, Deaths, and Case Fatality Rate by Province in Democratic Republic of Congo Between 1 January and 5 August 2024 [4]

Rank	Province	Suspected Cases	Confirmed Cases	Deaths	Case Fatality Rate, %
1	Equateur	5860	711	315	5.4
2	South Kivu	4173	1258	26	0.6
3	Sankuru	1566	63	60	3.8
4	South Ubangi	1398	142	33	2.4
5	Tshopo	769	202	32	4.2
6	Tshuapa	713	193	34	4.8
7	Mongala	668	104	25	3.7
8	Mai-Ndombe	276	119	10	3.6
9	North Kivu	249	64	0	0.0
10	Maniema	223	60	1	0.4
11	North Ubangi	121	7	5	4.1
12	Kasai	98	3	0	0.0
13	Lower Uele	92	4	3	3.3
14	Kinshasa	76	16	2	2.6
15	Upper Uele	25	1	1	4.0
16	Kwilu	24	5	0	0.0
17	Kwango	23	4	0	0.0
18	Kongo Central	15	1	0	0.0
19	Lualaba	15	1	0	0.0
20	Upper Lomami	12	0	0	0.0
21	Tanganyika	10	0	0	0.0
22	East Kasai	6	1	0	0.0
23	Kasai-Central	5	0	0	0.0
24	Ituri	4	0	0	0.0
25	Lomami	3	0	0	0.0
26	Upper Katanga	2	0	0	0.0

## ESCALATING CHALLENGES, WORSENED BY THE EMERGENCE OF A NEW VARIANT

The emergence of clade Ib, which is more transmissible than clade Ia, has worsened the outbreak situation [6]. Differences in pathogenicity, severity, risk groups, and transmission modes between clades Ib, Ia, and IIb complicate outbreak management [7]. Clade IIb, responsible for the 2022 multicountry outbreak, primarily spread via human-to-human contact, with 98% of cases among men aged 31–40 years for age, 96% of whom identified as men who have sex with men [8]. In contrast, clade Ia outbreaks in the DRC involve more diverse transmission, impacting all age groups and genders, with notable cases in children younger than 15 years, through human contact, zoonotic transmission, and environmental exposure (Table 2) [9]. Clade Ib, like clade IIb, spreads through human-to-human contact, affecting people aged 20–30 years, both men and women, likely via sexual transmission [3, 4]. Both clade Ia and clade Ib have a high case fatality rate of 1.8% [3], compared to 0.2% in clade IIb [10], posing significant public health challenges.

**Table 2.** Mpox Suspected Cases, Deaths, Case Fatality Rate, and Odd Ratio in Different Age Groups in Democratic Republic of Congo, 1 January to 5 August 2024 [4]

Age Group	Suspected Cases, No. (%)	Deaths, No. (%)	Case Fatality Rate, %	Crude OR (95% CI)	*P* Value
0–11 mo	1376 (9.3)	86 (16.0)	6.3	3.5 (2.6–4.7)	<.001
12–59 mo	3924 (26.5)	211 (39.3)	5.4	2.9 (2.3–3.7)	<.001
5–15 y	4055 (27.4)	137 (25.5)	3.4	1.8 (1.4–2.3)	<.001
>15 y	5443 (36.8)	103 (19.2)	1.9	1	…
Total	14 798	537	3.6	…	…

Abbreviations: CI, confidence interval; OR, odds ratio.

Mpox epidemics typically used to occur in equatorial forests, where zoonotic sources include squirrels, monkeys, and pangolins [11]. Poor hygiene, overcrowding, and reliance on the forest resources increase outbreak risks [12]. However, clades IIb and Ib have shifted this dynamic, emerging now in urban areas where previously it was rare [13]. Genomic surveillance of clade Ib identified APOBEC3-type mutations supporting human-to-human transmission [13]. While people with multiple sex partners and close contact with infected individuals are at higher risk, clade Ib can infect anyone in close contact [14]. The exact contribution of each transmission route remains unclear.

First reported in South Kivu in mid-September 2023, clade Ib has spread at least to 6 provinces in the DRC—South Kivu, North Kivu, Kinshasa, Kasai, Tshopo, and Tanganyika—by 3 November 2024 [3]. South Kivu, with the highest number of cases, appears to be stabilizing, while other provinces show mixed trends driven by localized hotspots. Testing challenges hinder accurate assessment of evolution of the outbreak. Its presence in international hubs like Kinshasa (DRC), Bujumbura (Burundi), and Kampala (Uganda) raises concerns about global spread [3].

As of 3 November 2024, 11 148 clade Ib cases have been reported globally, with the highest number of confirmed cases reported in the DRC (8662 cases, 77.7 %), followed by Burundi (1726, 15.5%) and Uganda (359, 3.2%) [3]. Other African countries reporting clade Ib cases include Rwanda (26 cases), Kenya (14 cases), Zimbabwe (2 cases), and Zambia (1 case). Outside Africa, cases have been reported in the United Kingdom (4 cases), and 1 each in Sweden, Thailand, India, Germany, Canada, and the United States [3, 15]. Community transmission is ongoing in Burundi and Uganda, with smaller clusters in Kenya and Rwanda. Cases in Zambia, Zimbabwe, and outside Africa are travel related. Notably, a clade Ib cluster in South Kivu involved children younger than 5 years, highlighting the need for further research on the strain's pathogenicity [3, 4]. Internationally, most cases are linked to sexual contact among young individuals, with transmission likely shifting to households and communities, increasing cases among children [3]. The growing clade Ib outbreak in the DRC and its international spread have raised global public health concerns. On 13 August 2024, the African Union Centers for Disease Control (Africa CDC) declared it a Public Health Emergency of Continental Security [16], followed by the World Health Organization (WHO) designating it as a Public Health Emergency of International Concern the next day [6].

## COORDINATED RESPONSE, SUPPORT, AND STRATEGIC MANAGEMENT

Coordinated efforts between governments, international organizations (eg, WHO, Africa CDC), academia, and local nongovernmental organizations are essential for efficient resource allocation and a unified response [6]. Collaboration with research consortia and humanitarian groups ensures effective specimen collection and continuous delivery of vital resources like food, vaccines, and protective equipment. Strategic planning and resource deployment are key to controlling the outbreak. Cross-border cooperation is vital to prevent spread, and mpox control should be integrated with broader infectious disease programs and surveillance systems for long-term sustainability [7].

Resource-rich countries can play a critical role by sharing resources, knowledge, and vaccines to support outbreak control. Measures like targeted vaccination, antiviral treatments, and health messaging helped contain the 2022 outbreak, but vaccine distribution remains inadequate, particularly for frontline workers and at-risk populations in the DRC [4]. Low- and middle-income countries (LMIC) face significant challenges due to weak infrastructure, limited resources, and poor legal frameworks, requiring international support [7]. Poor sanitation, malnutrition, and weakened immune systems increase vulnerability, especially in children and older adults [17]. Limited surveillance and incomplete data also challenge outbreak management, often needing external assistance [6, 18].

## NATIONAL MITIGATION PLAN IN DRC

The DRC's national mpox response plan strengthens multisectoral coordination under the One Health framework, integrating human, animal, and environmental surveillance [4]. It ensures medical care, province zoning, hospital district management, and food support for confirmed cases [4]. The plan emphasizes risk communication, addressing sexual transmission and human immunodeficiency virus (HIV)-mpox coinfection, and prioritizes vaccination for high-risk populations. It also focuses on infection prevention, better laboratory diagnostics, psychological support, and sufficient resources for incident management [4]. However, it lacks environmental surveillance actions, which could improve mpox detection, track zoonotic transmission, and fill a critical gap in the response strategy.

## AFRICA CDC PROTOCOL FOR MPOX SURVEILLANCE AND REPORTING

Africa CDC has published a mpox surveillance and reporting protocol for African Union member countries [19]. The protocol aims to establish a robust, standardized, and coordinated surveillance approach, enabling early detection and prompt response. It seeks to strengthen existing surveillance systems by enhancing efforts in communities, healthcare facilities, and points of entry, while linking these activities to national and regional laboratories for case confirmation [19]. The protocol outlines a comprehensive strategy to improve the detection and monitoring of mpox cases, focusing on improved data collection, analysis, genomic sequencing, and cross-border coordination to expedite outbreak response [19].

## WASTEWATER AND ENVIRONMENTAL SURVEILLANCE COULD ADDRESS GAPS IN CURRENT MONITORING EFFORTS

Given the virulence of clade Ib, robust surveillance—tracking travel history, contact, behavioral risks, health conditions, and vaccination status—is vital for containment and contact tracing [20]. However, clinical reporting faces challenges like limited testing, stigma linked to sexually transmitted diseases, fear of isolation, and undiagnosed or asymptomatic cases [21]. Wastewater surveillance (WWS) could serve as a solution for these matters, and additionally contribute to genomic surveillance, which is vital for tracking variants and identifying risk factors [22].

Individuals with mpox infection shed high viral loads through body fluids like urine, saliva, semen, feces, and lesion exudates, in both symptomatic and asymptomatic individuals, making WWS a promising tool [23]. In endemic regions, WWS can identify variants and clades via polymerase chain reaction (PCR) or metagenomics, providing insights into mpox's spatial and temporal patterns [23]. It has proven effective in detecting outbreaks early, offering cost-effective, near real-time data on disease trends, often preceding symptom onset and medical care [22]. This early detection is crucial in countries like the DRC, where underreporting is common due to limited testing [5]. Nonhuman primates and rodents, suspected reservoirs, are often excluded from surveillance. WES could detect virus circulation in these populations. Adopting a One Health approach, integrating human, animal, and environmental health, is crucial for comprehensive outbreak management [22].

Combining clinical and environmental surveillance strengthens outbreak response, although WES faces challenges like low pathogen titers and PCR inhibitors [24]. In LMIC, the lack of centralized sewage systems hinders sampling and surveillance outcomes [24]. Understanding variant-specific shedding dynamics is key for accurate monitoring. While WWS works well in areas with centralized sewage, in many LMIC the lack of infrastructure may require sampling of grey water in nonsewered areas rather than relying on urban rivers, pit latrines, or septic tanks [25].

## CONCLUSION

The surge of mpox clade Ib poses a significant outbreak risk due to its high pathogenicity. Various key aspects, like transmission, severity, risk factors, and diagnostic challenges, remain poorly understood, highlighting the need for further research. While the One Health approach has been effective, WES remains underutilized. Integrating it into current strategies is paramount.

## Notes

***Author contributions.*** A. T. conceptualized and drafted the initial manuscript. T. K. and V. M. provided materials and data from the Democratic Republic of Congo. AK, MCT, RL, TP, and VM secured funding. All authors contributed to the draft and approved the final submitted version of the manuscript.

***Disclaimer***. All the claims expressed in this article are solely those of the authors and do not necessarily represent those of their affiliated organizations or those of the publisher, the editors, or the reviewers. It neither the European Union nor the funding authority can be held responsible for them.

***Financial support.*** This work was supported by the European Union (grant number 101195186 ODIN-Mpox-HORIZON-JU-GH-EDCTP3-2024-Mpox). T. V. D. B. acknowledges funding from the Research Foundation Flanders (grant number 1286824N).
